# Comprehensive evaluation of clinical outcomes in hepatic epithelioid hemangioendothelioma subsets: insights from SEER Database and departmental cohort analysis

**DOI:** 10.3389/fimmu.2024.1491922

**Published:** 2024-10-22

**Authors:** Bingchen Wang, Xiao Chen, Rongxuan Li, Bolun Ai, Feng Ye, Jianjun Zhao, Yefan Zhang, Zhen Huang, Zhiyu Li, Xinyu Bi, Hong Zhao, Dayong Cao, Jianqiang Cai, Jianguo Zhou, Tao Yan

**Affiliations:** ^1^ Department of Hepatobiliary Surgery, National Cancer Center/National Clinical Research Center for Cancer/Cancer Hospital, Chinese Academy of Medical Sciences and Peking Union Medical College, Beijing, China; ^2^ Department of Breast Surgical Oncology, National Cancer Center/National Clinical Research Center for Cancer/Cancer Hospital, Chinese Academy of Medical Sciences and Peking Union Medical College, Beijing, China; ^3^ Department of Radiology, National Cancer Center/National Clinical Research Center for Cancer/Cancer Hospital, Chinese Academy of Medical Sciences and Peking Union Medical College, Beijing, China; ^4^ Department of Anesthesiology, National Cancer Center/National Clinical Research Center for Cancer/Cancer Hospital, Chinese Academy of Medical Sciences and Peking Union Medical College, Beijing, China

**Keywords:** general surgery, hepatic epithelioid hemangioendothelioma, SEER, Cox regression analyses, machine learning

## Abstract

**Background:**

Epithelioid hemangioendothelioma (EHE), is an uncommon, intermediate-grade malignant vascular tumor that can manifest in diverse organs, including the liver, lungs, and bones. Given its unique malignancy profile and rarity, there lacks a consensus on a standardized treatment protocol for EHE, particularly for hepatic epithelioid hemangioendothelioma (HEHE). This study aims to elucidate factors influencing the clinical prognosis of EHE by analyzing data from the SEER database, complemented with insights from a departmental cohort of 9 HEHE cases. Through this, we hope to shed light on potential clinical outcomes and therapeutic strategies for HEHE.

**Methods:**

Using SEER data from 22 registries, we analyzed 313 liver cancer patients with ICD-O-3 9130 and 9133 histology. Twelve variables were examined using Cox regression and mlr3 machine learning. Significant variables were identified and compared. Clinical data, imaging characteristics, and treatment methods of nine patients from our cohort were also presented.

**Result:**

In univariate and multivariate Cox regression analyses, Age, Sex, Year of diagnosis, Surgery of primary site, Chemotherapy, and Median household income were closely related to survival outcomes. Among the ten survival-related machine learning models, CoxPH, Flexible, Mboost, and Gamboost stood out based on Area Under the Curve(AUC), Decision Curve Analysis(DCA), and Calibration Curve Metrics. In the feature importance analysis of these four selected models, Age and Surgery of primary site were consistently identified as the most critical factors influencing prognosis. Additionally, the clinical data of nine patients from our cohort not only demonstrated unique imaging characteristics of HEHE but also underscored the importance of surgical intervention.

**Conclusion:**

For patients with resectable HEHE, surgical treatment is currently a highly important therapeutic approach.

## 1 Introduction

Epithelioid hemangioendothelioma (EHE) is a rare vascular tumor, first high-lighted by the seminal studies of Weiss and Enzinger in 1982 (1). This low-grade malignancy is characterized by its unique assembly of predominantly epithelioid endothelial cells. While EHE can be found in various anatomical locations, its presence in the liver, known as hepatic epithelioid hemangioendothelioma (HEHE), often navigates the challenging waters of diagnosis, sometimes being mistaken for other hepatic tumors (2, 3). Intriguingly, epidemiological insights suggest a greater inclination toward females, especially those aged 40-55, though its overall incidence is notably low, less than 0.1 per 100,000 (3–9). A defining characteristic of EHE is its absence of vasoformation, distinguishing it from other vascular tumors (10). Delving into its molecular underpinnings, chromosomal rearrangements involving the WWTR1 and CAMTA1 genes emerge as key players, complemented by the noteworthy YAP-THE3 gene fusion (11–13). Diagnostically, EHE’s marked preference for specific endothelial markers is a pivotal feature (14).

Clinical presentations of Hepatic Epithelioid Hemangioendothelioma (HEHE) are diverse. While some patients exhibit no symptoms, others may experience a range of manifestations, including right upper quadrant pain, weight loss, jaundice, nausea, anorexia, fatigue, and hepatomegaly (3, 4, 6, 9). In radiographic evaluations, the nodules of HEHE routinely appear to be multiple and peripheral in the image presentation. HEHE distinctly manifests through three primary characteristics: the Capsular Retraction (15, 16), indicative of liver tissue hypertrophy due to tumor-associated fibrotic changes; the Target Sign on T2W imaging, epitomized by a central high-intensity core, flanked by a low-intensity ring and subtly accentuated by an outer high-intensity halo (17); and the Lollipop Sign in enhanced imaging, where the ‘candy’ delineates the evident tumor mass, while the ‘bar’ depicts the occluded vein on T2WI (18). Collectively, these imaging signatures are instrumental in differentiating HEHE from other hepatic metastatic entities.

Due to the rarity of EHE and limited research available, a standardized treatment protocol for HEHE has yet to be established. For 253 diagnosed HEHE patients, survival rates irrespective of the treatment approach were observed to be 83.4% (211 patients) at 1 year, 55.7% (141 patients) at 3 years, and 41.1% (104 patients) at 5 years (9). For HEHE patients, various treatment modalities have been explored in clinical trials and analyses. These include surgical options like hepatectomy and liver transplantation (LT), alternative therapies such as ablation and transcatheter arterial chemoembolization (TACE), as well as systemic treatments encompassing chemotherapy, anti-VEGF therapy, and mTOR inhibitors (19–22). However, one study suggested that the 5-year survival rates across various treatment approaches showed no significant differences, leading to a recommendation for a watchful waiting strategy (23). While the mechanisms and progress in basic research on EHE are continuously advancing, surgical treatments remain the most common and major treatments for patients with EHE at present from a clinical aspect (9).

## 2 Materials and methods

### 2.1 The data sources

From the SEER Research data encompassing 22 registries, 313 liver cancer patients diagnosed with histology ICD-O-3 9133 (EHE) and 9130 (HE) were identified for detailed analysis. Key variables such as age, sex, year of diagnosis, race, combined summary stage, surgery of primary site, radiotherapy, chemotherapy, preoperative or postoperative systemic therapy, sequence number, median household income, rural urban continuum code, survival duration, and living status were extracted from their respective fields in the SEER database.

For this study, 9 HEHE patients were selected from our hospital. The inclusion criteria were: 1) patients who underwent liver resection due to hepatic lesions between 2012 and 2022 and were pathologically diagnosed with HEHE post-surgery, and 2) patients aged 18 years or older. Patients were excluded from the study if they met any of the following criteria: 1) Patients who did not undergo surgical resection, and 2) Patients under 18 years of age. Detailed baseline information was compiled, including gender, age, primary diagnosis, tumor ICD, clinical symptoms, underlying conditions, and physical signs. Radiological features covered tumor location, size, multiplicity, peripheral involvement, capsular retraction, target sign, and lollipop sign. Pathological features, including immunohistochemical markers like CD34, CD31, ERG, and Fli1, were documented, with results presented in an embedded pie chart. Laboratory results included markers such as CA199, AFP, CEA, ALT, AST, and additional tests. All clinical and pathological data were obtained through routine hospital procedures. This study was approved by the Institutional Review Board of the Cancer Hospital, Chinese Academy of Medical Sciences and Peking Union Medical College. All patients provided informed consent.

### 2.2 Statistical analysis

**Figure 1:**
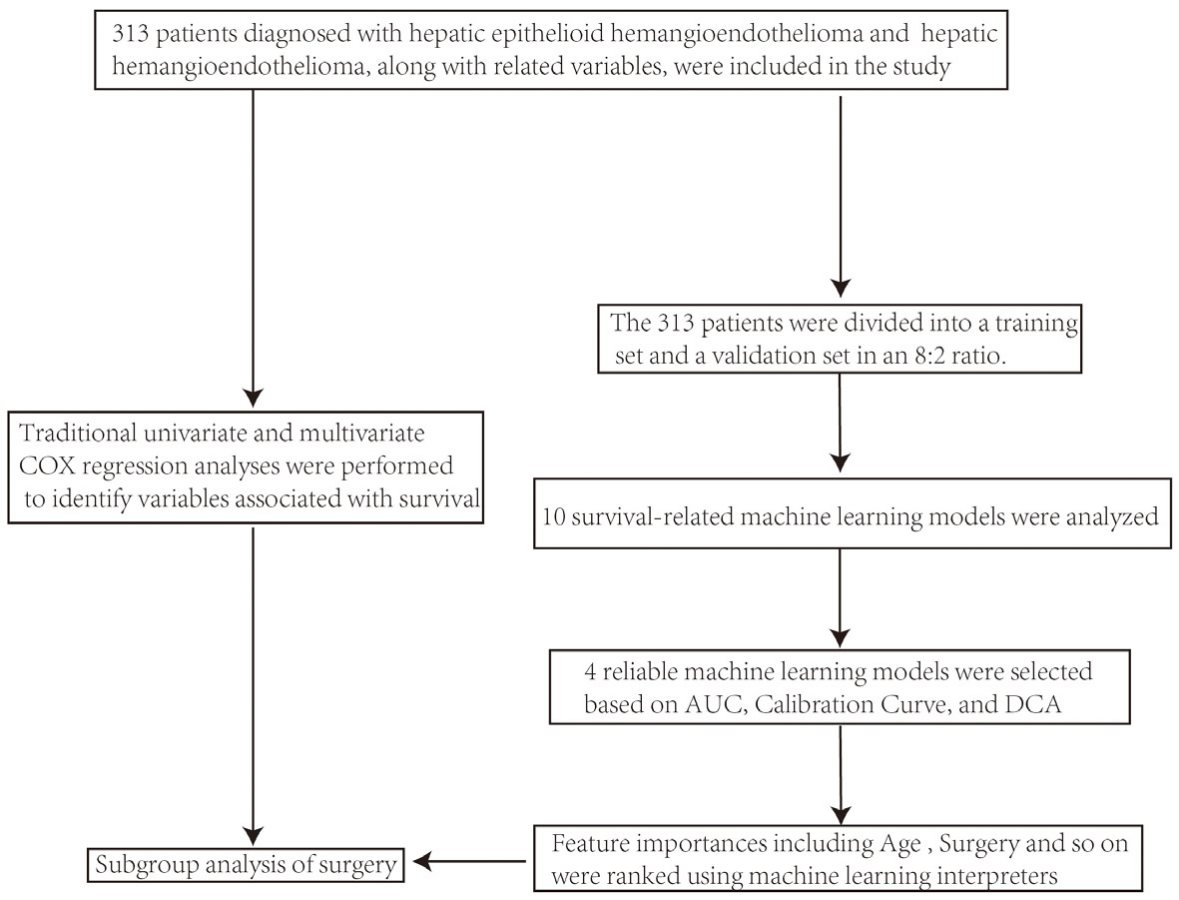
Flowchart of analysis using the SEER database.

Statistical analyses were primarily executed in R Studio. Using the autoReg package, we performed univariate and multivariate Cox regression analyses to identify variables related to survival. The dataset was divided into training and validation sets in an 8:2 ratio to prepare for survival-related machine learning and there were no statistically significant differences in baseline characteristics (**Supplementary Table 1**) or survival information (**Supplementary Figure 1**) between the training and validation sets. We constructed survival-related machine learning models using the following mlr3 models: “surv.coxph” (a traditional proportional hazards model), “surv.cv_glmnet” (a regularized regression model), “surv.rpart” (a decision tree-based model), “surv.rfsrc” (a random forest model), “surv.gbm” (a gradient boosting model), “surv.flexible” (a flexible parametric spline learner using flexsurv::flexsurvspline()), “surv.blackboost”, “surv.gamboost”, and “surv.glmboost” (all boosting-based models). For each model, we calculated and plotted the AUC (evaluating discriminatory power), calibration curves (assessing prediction accuracy), and DCA (analyzing clinical utility) for both the training and validation sets. Models with an AUC above 0.75, calibration curves fitting the reference line, and beneficial DCA were selected. Feature importance analysis was conducted on the top-performing models to rank variables influencing survival. The important variables identified by traditional Cox regression and machine learning (especially treatment methods) were further analyzed in different subgroups (**Figure 1**).

## 3 Results

### 3.1 Study population and Cox regression

The baseline characteristics of 313 patients, stratified by the surgery of the primary site, are presented in **Table 1**. It can be observed that the choice of surgery of the primary site among the various groups shows statistically significant differences only in the preoperative or postoperative systemic therapy group. In all other groups, the surgery of the primary site (including no surgery or unknown, wedge or segmental resection, lobectomy, hepatectomy, and transplant) does not exhibit statistically significant differences.

**Table 1:** Baseline Characteristics of 313 Patients Stratified by Primary Site Surgery

	no surgery or unknown	Wedge or segmental resection or Lobectomy	Hepatectomy and or transplant	p.overall
	*N=229*	*N=58*	*N=26*	
Age	55.0 [39.0;65.0]	53.5 [37.2;62.8]	45.0 [38.2;54.2]	0.164
Sex:				0.301
Male	107 (46.7%)	23 (39.7%)	15 (57.7%)	
Female	122 (53.3%)	35 (60.3%)	11 (42.3%)	
Year of diagnosis:				0.454
before and in 2010	106 (46.3%)	23 (39.7%)	14 (53.8%)	
after 2010	123 (53.7%)	35 (60.3%)	12 (46.2%)	
Race:				0.858
White	183 (79.9%)	48 (82.8%)	22 (84.6%)	
others	46 (20.1%)	10 (17.2%)	4 (15.4%)	
Combined Summary Stage:				
Localized	49 (21.4%)	34 (58.6%)	6 (23.1%)	
Regional	25 (10.9%)	9 (15.5%)	12 (46.2%)	
Distant	91 (39.7%)	11 (19.0%)	6 (23.1%)	
Unknown/unstaged	64 (27.9%)	4 (6.90%)	2 (7.69%)	
Radiation recode:				0.450
None or Unknown	221 (96.5%)	56 (96.6%)	24 (92.3%)	
radiation performed	8 (3.49%)	2 (3.45%)	2 (7.69%)	
Chemotherapy recode:				0.120
No or Unknown	161 (70.3%)	48 (82.8%)	17 (65.4%)	
Yes	68 (29.7%)	10 (17.2%)	9 (34.6%)	
Systemic Sur Seq:				<0.001
no Systemic therapy after and before surgery	225 (98.3%)	50 (86.2%)	19 (73.1%)	
Systemic therapy after or before surgery	4 (1.75%)	8 (13.8%)	7 (26.9%)	
Sequence number:				0.806
One primary only	187 (81.7%)	47 (81.0%)	20 (76.9%)	
Over one	42 (18.3%)	11 (19.0%)	6 (23.1%)	
Median_household_income_inflation_adj_to_2021:				0.807
below $70000	113 (49.3%)	26 (44.8%)	12 (46.2%)	
more than $70000	116 (50.7%)	32 (55.2%)	14 (53.8%)	
Rural Urban Continuum Code:				0.441
Metropolitan (1 million+)	161 (70.3%)	37 (63.8%)	20 (76.9%)	
Other metropolitan or non-metropolitan	68 (29.7%)	21 (36.2%)	6 (23.1%)	

**Table 2** also presents data for the 313 patients, showing that the mean age at diagnosis is 51 years, with a higher proportion of female patients (53.7%). A portion of the patients (34.5%) were diagnosed at the distant stage. Univariate and multivariate Cox regression analyses identified age (Final HR: 1.03 [1.02-1.04], p<.001), gender (Female vs. Male, Final HR: 0.67 [0.48-0.93], p=.017), year of diagnosis (after 2010 vs. before 2010, Final HR: 0.53 [0.37-0.75], p<.001), surgery of the primary site (wedge or segmental resection or lobectomy vs. no surgery or unknown, Final HR: 0.43 [0.25-0.73], p=.002; hepatectomy and/or transplant vs. no surgery or unknown, Final HR: 0.27 [0.11-0.66], p=.004), chemotherapy (yes vs. no or unknown, Final HR: 1.89 [1.32-2.72], p=.001), and median household income (more than $70,000 vs. below $70,000, Final HR: 0.57 [0.41-0.80], p=.001) as significant variables influencing survival prognosis.

**Table 2:** Univariate and Multivariate Cox Regression Analyses

	Dependent: Surv(time, status == 1)	all	HR (univariable)	HR (multivariable)	HR (final)
Age	Mean ± SD	51.0 ± 17.9	1.03 (1.02-1.04, p<.001)	1.04 (1.02-1.05, p<.001)	1.03 (1.02-1.04, p<.001)
Sex	Male	145 (46.3%)			
	Female	168 (53.7%)	0.69 (0.49-0.95, p=.025)	0.68 (0.49-0.95, p=.024)	0.67 (0.48-0.93, p=.017)
Year of diagnosis	before and in 2010	143 (45.7%)			
	after 2010	170 (54.3%)	0.68 (0.48-0.96, p=.027)	0.59 (0.41-0.86, p=.006)	0.53 (0.37-0.75, p<.001)
Race	White	253 (80.8%)			
	others	60 (19.2%)	1.12 (0.75-1.69, p=.576)		
Combined Summary Stage	Localized	89 (28.4%)			
	Regional	46 (14.7%)	1.31 (0.72-2.37, p=.374)	1.39 (0.75-2.57, p=.293)	
	Distant	108 (34.5%)	1.86 (1.16-2.96, p=.010)	1.51 (0.90-2.54, p=.116)	
	Unknown/unstaged	70 (22.4%)	2.09 (1.28-3.40, p=.003)	1.88 (1.08-3.30, p=.026)	
Surg Prim Site	no surgery or unknown	229 (73.2%)			
	Wedge or segmental resection or Lobectomy	58 (18.5%)	0.41 (0.24-0.69, p<.001)	0.54 (0.31-0.96, p=.036)	0.43 (0.25-0.73, p=.002)
	Hepatectomy and or transplant	26 (8.3%)	0.26 (0.11-0.64, p=.003)	0.29 (0.12-0.73, p=.009)	0.27 (0.11-0.66, p=.004)
Radiation recode	None or Unknown	301 (96.2%)			
	radiation performed	12 (3.8%)	1.85 (0.90-3.77, p=.092)		
Chemotherapy recode	No or Unknown	226 (72.2%)			
	Yes	87 (27.8%)	1.53 (1.08-2.17, p=.017)	2.00 (1.34-2.99, p=.001)	1.89 (1.32-2.72, p=.001)
Systemic Sur Seq	no Systemic therapy after and before surgery	294 (93.9%)			
	Systemic therapy after or before surgery	19 (6.1%)	0.94 (0.48-1.85, p=.857)		
Sequence number	One primary only	254 (81.2%)			
	Over one	59 (18.8%)	1.40 (0.95-2.06, p=.091)		
Median_household_income_inflation_adj_to_2021	below $70000	151 (48.2%)			
	more than $70000	162 (51.8%)	0.68 (0.49-0.95, p=.023)	0.56 (0.40-0.79, p=.001)	0.57 (0.41-0.80, p=.001)
Rural Urban Continuum Code	Metropolitan (1 million+)	218 (69.6%)			
	Other metropolitan or non-metropolitan	95 (30.4%)	1.37 (0.97-1.93, p=.070)		

n=313, events=143, Likelihood ratio test=95.49 on 10 df(p<.001).

### 3.2 Construction and selection of survival-related machine learning models and presentation of variable importance

**Figure 2:**
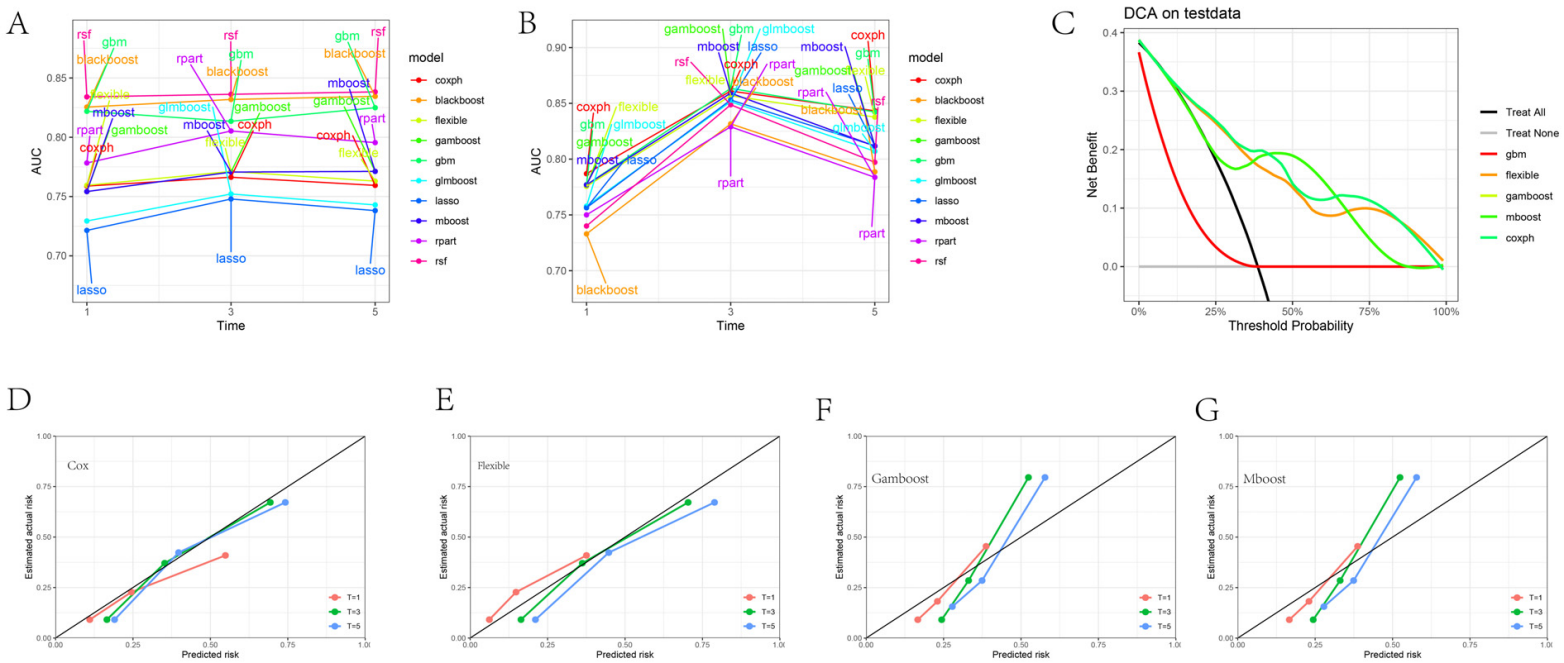
Machine Learning Model Evaluation and Display. **(A)** AUC values of 10 different machine learning models at 1, 3, and 5-year specific time points in the training set. **(B)** AUC values of 10 different machine learning models at 1, 3, and 5-year specific time points in the test set. **(C)** DCA plot of selected machine model. **(D–G)** Calibration plot of CoxPH, Flexible, Gamboost, mboost machine model.

**Figure 3:**
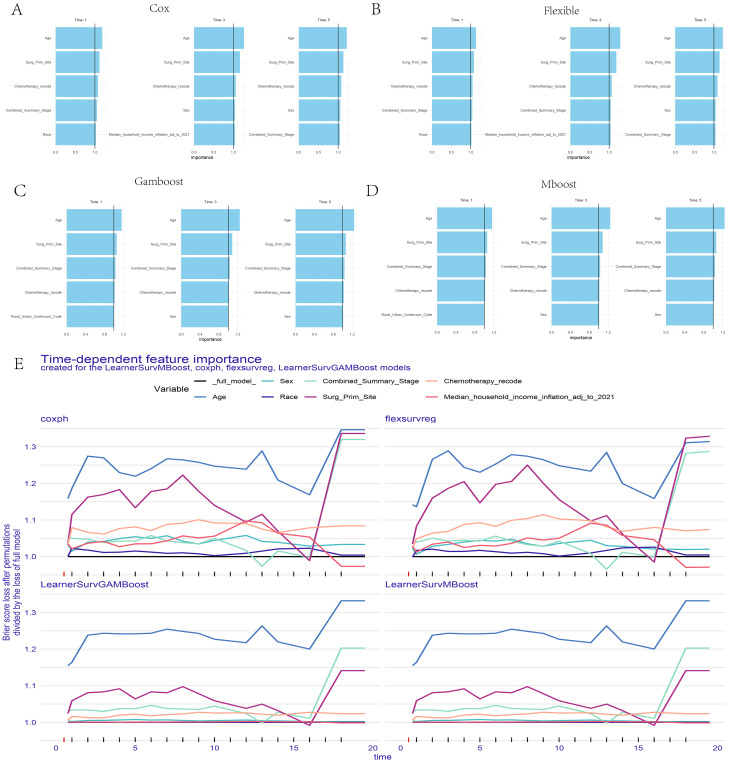
Feature Importance Ranking Display of the Four Selected Models. **(A)** CoxPH model. **(B)** Flexible model. **(C)** Gamboost model **(D)** Mboost model **(E)** Time-dependent feature importance of the four models.

**Figure 2A** presents the AUC values of 10 different machine learning models at 1, 3, and 5-year specific time points in the training set, while **Figure 2B** shows the AUC values for these models at the same time points in the validation set. It was observed that the AUC values of the CoxPH model, flexible model, gamboost model, mboost model, and gbm model were all greater than 0.75 (**Supplementary Figures 2A–J**). However, the DCA curve indicated that the performance of the gbm model was not ideal (**Figure 2C**), whereas the calibration curves for the CoxPH model, flexible model, gamboost model, and mboost model fit the reference line (**Figures 2D–G**). Consequently, the CoxPH model, flexible model, gamboost model, and mboost model were selected for subsequent variable importance analysis. **Figures 3A–D** illustrate the feature importance of relevant variables for the CoxPH model, flexible model, gamboost model, and mboost model at 1, 3, and 5 years, while **Figure 3E** presents the time-dependent variable importance. It can be clearly seen that, across the different time points, Age and surgery of the primary site consistently emerged as the two most significant factors influencing prognosis. Basic sequencing studies have also found that age is associated with rapid tumor progression, and surgical treatment remains a superior option in the absence of effective chemotherapy and targeted therapy.

### 3.3 Group analyses of surgery and comparison of surgery types

**Figure 4:**
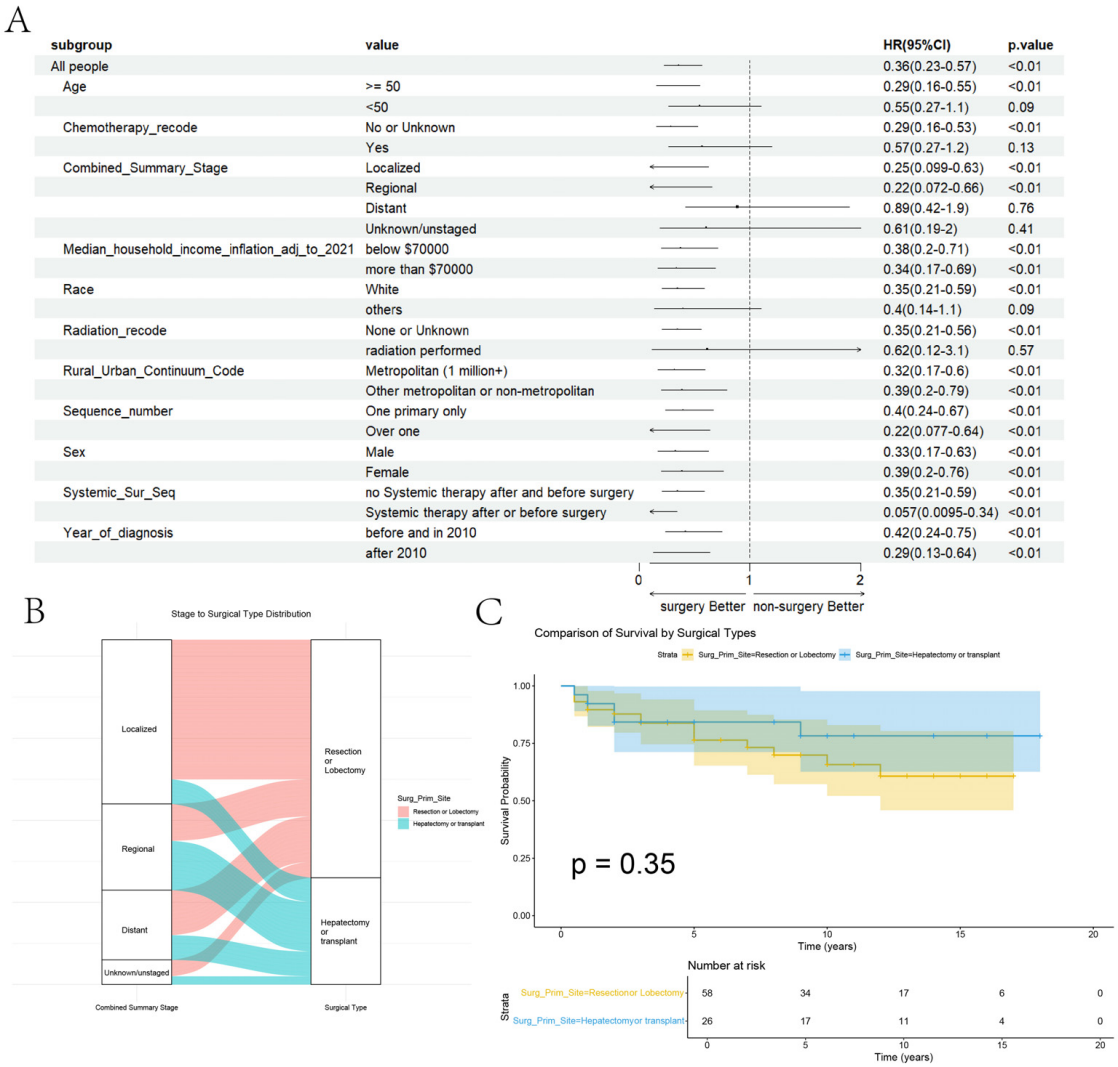
Group Analyses of Surgery and Comparison of Surgery Types. **(A)** Group Analyses of Surgery within the related variables. **(B)** The Sankey diagram illustrates the relationship between tumor staging and surgical approaches. **(C)** Comparison of the Kaplan-Meier curves between the two surgical approaches.

At the aim of further exploring the specific therapeutic role of surgery in HEHE tumors, group analyses of surgery’s presence or absence within “Age” group, “Chemotherapy” group, “Stage” group, “Median house income” group, “Race” group, “Radiotherapy” group, “Rural urban continuum” group, “Sequence number” group, “Sex” group, “Systemic therapy and surgery” group and “Year of diagnosis” group, are performed and the forest plot of group analyses is drawn (**Figure 4A**).

In the forest plot for subgroup analysis, it is evident that surgery has a positive effect across various groups, including the year of diagnosis, presence of preoperative or postoperative adjuvant therapy, gender, sequence number, rural-urban continuum code, and income. Patients with localized or regional stage disease benefit from surgery, whereas those with distant stage disease do not show a significant benefit. Patients undergoing chemotherapy or radiation therapy may not derive a clear benefit from surgery, likely due to their more advanced stage at diagnosis. Interestingly, patients younger than 50 years old do not seem to benefit from surgical treatment, which may be due to the limited sample size leading to the lack of statistical significance for surgical treatment. After performing propensity score matching (PSM) to ensure baseline comparability for surgery performed or not (**Supplementary Table 2**), surgery remained a significant variable associated with better prognosis in both univariate and multivariate Cox regression analyses (**Supplementary Table 3**). Overall, it can be concluded that patients with limited tumor stages who meet the surgical criteria should undergo further surgical treatment.

In terms of surgical approaches, no significant differences were observed between resection or lobectomy and hepatectomy or transplant across patient-level variables, apart from tumor staging (**Supplementary Table 4**). The Sankey diagram further highlights a preference for resection or lobectomy in patients with localized tumors, whereas hepatectomy or transplant was more frequently chosen for those with regional or distant disease (**Figure 4B**). Nonetheless, no statistically significant differences in survival outcomes were detected between the two surgical strategies (**Figure 4C**).

### 3.4 Clinical information for the nine patients from our departmental cohort

**Figure 5:**
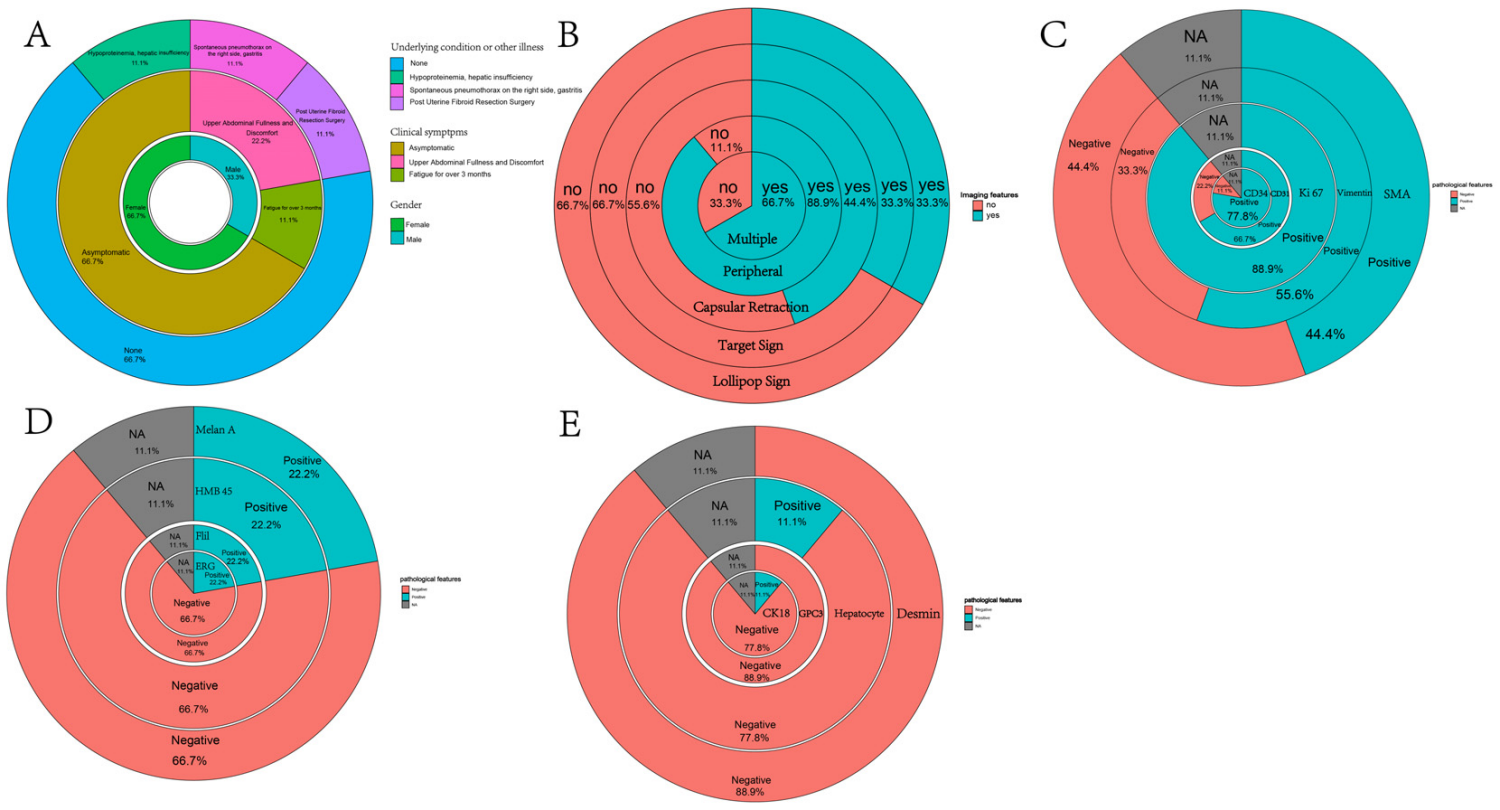
Inset pie charts visualizing various types of information about HEHE patients. **(A)** Inset pie chart showing the underlying condition or illness, clinical symptoms, and gender of 9 HEHE patients. **(B)** Inset pie chart showing the imaging features of 9 patients. **(C)** Inset pie chart showing pathological specimens of 9 patients with immunohistochemical information on CD34, CD31, Ki67, Vimentin, SMA. **(D)** Inset pie chart showing pathological specimens of 9 patients with immunohistochemical information on ERG, Flil, HMB45, MelanA. **(E)** Inset pie chart showing pathological specimens of 9 patients with immunohistochemical information on CK18, GPC3, Hepatocyte, Desmin.

**Figure 6:**
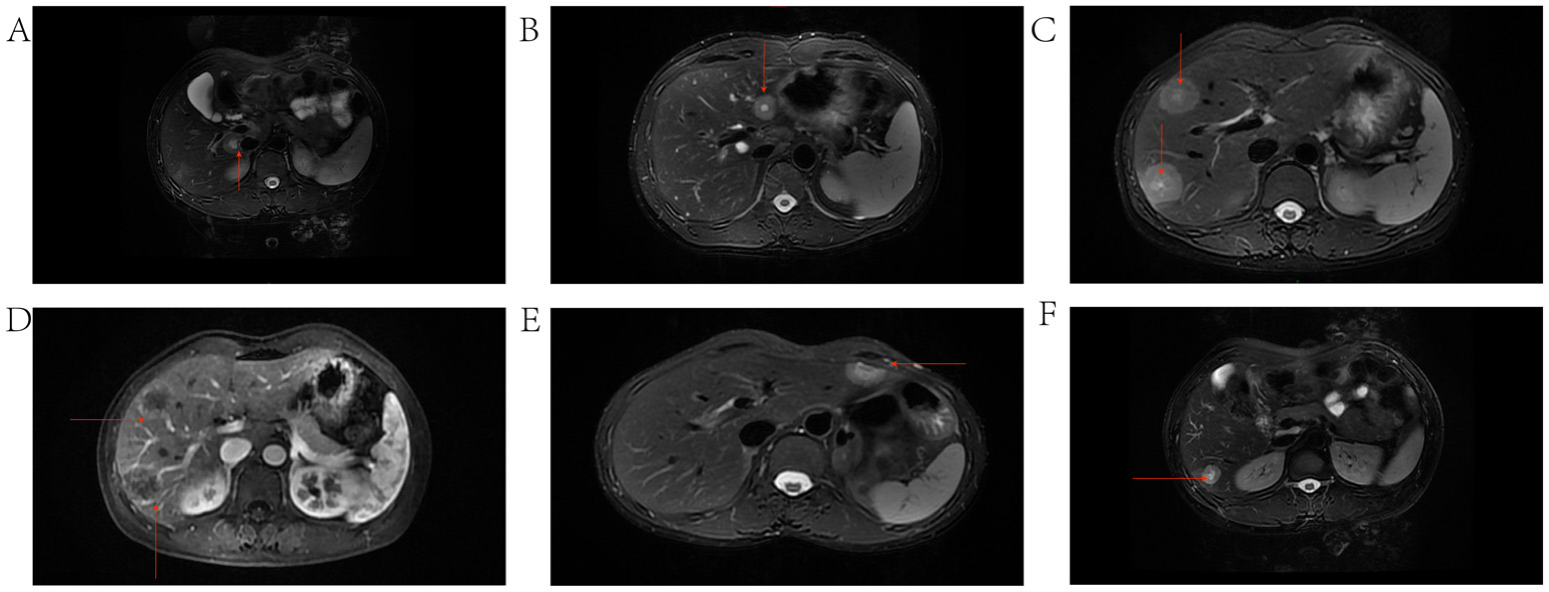
Typical imaging features including capsular retraction, ‘target’ and ‘lollipop’ signs on MRI. **(A)** HEHE tumor located in the periphery of the caudate lobe of the liver exhibiting the target sign and capsular retraction. **(B)** HEHE tumor located in the periphery of the left lobe of the liver exhibiting a Target Sign. **(C)** Multiple HEHE tumors located in the peripheral areas of segment VIII of the liver, exhibiting target signs. **(D)** EHE tumors exhibiting the Lollipop sign, where the tumor represents the head of the lollipop, and the tortuous, occluded vessels form the stick of the lollipop. **(E)** HEHE tumor located in the periphery of segment III of the liver, exhibiting a Target Sign. **(F)** HEHE tumor located in the periphery of the right lobe of the liver, exhibiting the target Sign.

**Figure 7:**
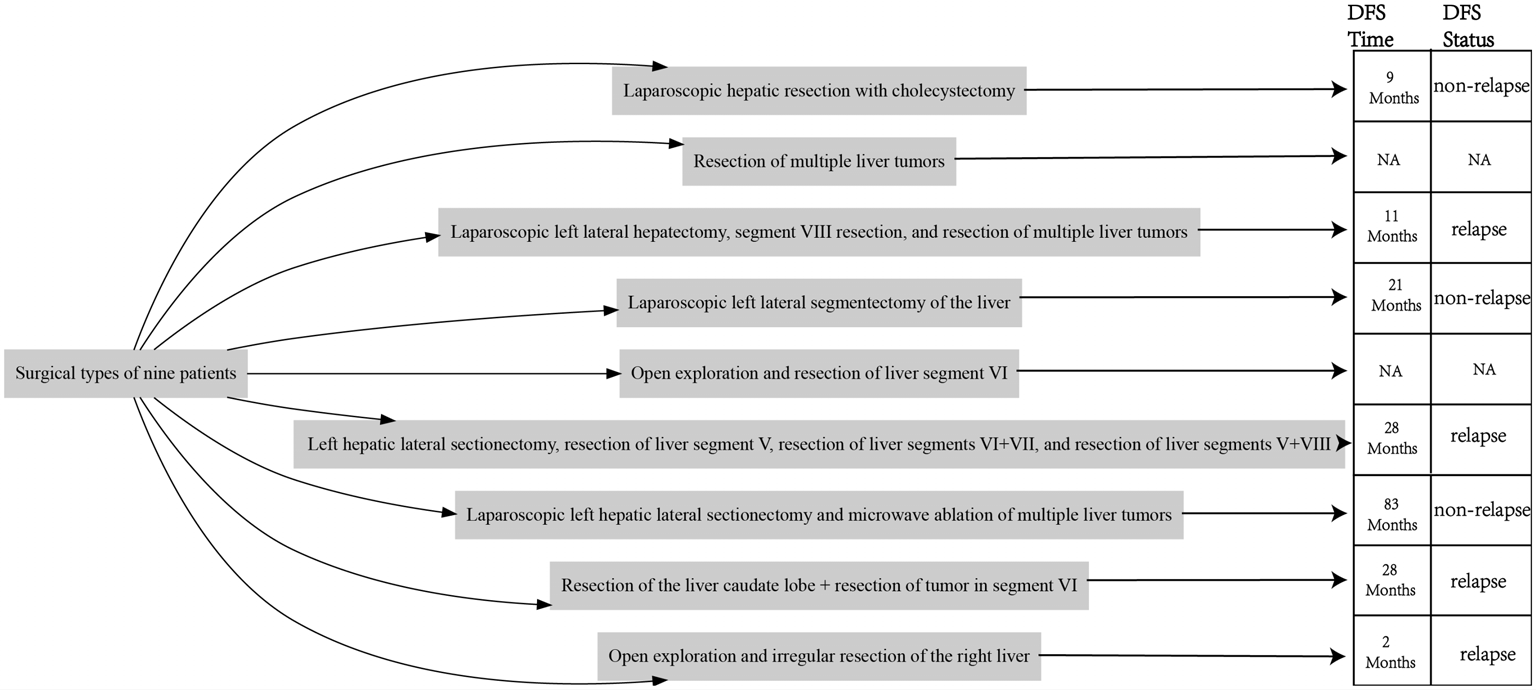
Surgery types and corresponding PFS information of the 9 HEHE patients.

Inset pie charts are used to visualize baseline information, image feature, pathological features about HEHE patients in our cohort (**Figure 5**). It’s evident that the majority of patients are in good overall health, with 66.7% having no concurrent illnesses. Similarly, 66.7% of the patients are asymptomatic, and a significant portion of the cohort is female (**Figure 5A**). When it comes to imaging information for HEHE patients, the majority of tumor lesions are characterized as multiple (66.7%) and peripheral (88.9%). Capsular retraction is observed in 44.4% of cases, while ‘target’ and ‘lollipop’ signs are also significant features, each present in 33.3% of the images (**Figures 5B**, **6**). It can be observed that peripheral ‘target’ signs in T2 MRI (**Figures 6A–C, E, F**) and capsular retraction (**Figure 6A**). ‘Lollipop’ signs can be clearly detected in which offer the valuable imaging feature (**Figure 6D**). The pathological features of HEHE samples from our department can be summarized as follows: HEHE tumors exhibit a range of sizes with some showing no cumulative liver involvement. Histologically, these tumors often present with epithelial-like or spindle-shaped cells, some of which have cellular atypia. Notably, features such as fatty degeneration of surrounding liver tissue, vacuoles in the cytoplasmic membrane, and rare nuclear mitoses can be observed. Furthermore, the presence of multinucleated cells and cells arranged in nests are consistent with epithelioid hemangioendothelioma morphology. When it comes to the pathological immunohistochemical features, the majority of specimens show positive staining for CD31 and CD34 (former), as well as for Ki67, Vimentin, and SMA (latter) at rates of 77.8%, 66.7%, 88.9%, 55.6%, and 44.4%, respectively. The former two markers signify endothelial characteristics, while the latter two indicate cytokeratin and smooth muscle actin markers. Ki67, in particular, implies the proliferative nature of the tumor cells (**Figure 5C**). A small portion of them also stains positively for MelanA, HMB 45, Flil, ERG in immunohistochemistry while few of them stains positively for CK18, GPC3, Hepatocyte, Desmin (**Figures 5D, E**). The laboratory parameters (mainly including liver function and tumor markers) of the 9 patients before and after surgery are mostly within the normal range. All nine EHE patients underwent surgery corresponding to the site of tumor growth and their progression-free survival (PFS) was listed (**Figure 7**). The HEHE recurrence rate is relatively high (4/9),with nearly all recurrence sites located in adjacent liver tissues. Due to the small sample size, it is not sufficient to draw statistically significant conclusions, and further relevant analysis cannot be conducted.

## 4 Discussion

Epithelioid hemangioendothelioma (EHE) is a unique vascular tumor, initially identified in 1982. When it appears in the liver, it’s termed hepatic epithelioid hemangioendothelioma (HEHE) and often poses diagnostic challenges. Though prevalent in females aged 40-55, its incidence is low. Distinctive molecular markers and its absence of vasoformation set it apart from similar tumors. Clinically, HEHE symptoms vary, with some patients being asymptomatic, while others show signs like pain and weight loss. Radiographically, HEHE is characterized by its peripheral distribution and multifocality. Additionally, distinctive imaging features include capsular retraction, ‘target’ sign, and ‘lollipop’ signs. Treatment protocols are diverse and not yet standardized due to its rare occurrence.

Despite the limited dataset in our department, which consists of only 9 cases, the clinical characteristics of HEHE patients exhibit a certain degree of representativeness. Additionally, by combining our departmental data with existing literature, certain clinical features of HEHE can be elucidated. In HEHE patients, a preference for females, non-specific clinical symptoms, and the presence of multiple and peripheral nodular lesions are main manifestations. Capsular retraction, along with the ‘target’ and ‘lollipop’ signs, are prominent radiological hallmarks observed in HEHE imaging. However, right upper quadrant pain is regarded as the most common clinical manifestation of HEHE (5, 6) and some rare cases had rare syndrome such as Budd-Chiari syndrome (24) and Kasabach–Merrit syndrome (25). On contrast-enhanced study, CT and MRI share common features and the character can be described as three patterns. Some tumors display mild homogeneous enhancement in arterial phase without any change in the delayed or portal vein phase. Some masses show ring like enhancement at first in the arterial phase and full enhancement in the delayed and portal phases, which is called “halo sign”. And the last type is the heterogeneous enhancement which progresses in all phases (26, 27). It has been concisely summarized that lesions smaller than 2 cm predominantly exhibit mild homogeneous enhancement; lesions ranging from 2-3cm display ring-like enhancement transitioning to heterogeneous delayed enhancement; lesions exceeding 3 cm predominantly manifest heterogeneous delayed enhancement (28, 29). The imaging characteristics of our cohort of 9 patients, especially those discerned from MRI, largely resonate with the imaging findings delineated in the review literature concerning EHE patients. However, the manifestations of enhancement types in HEHE may seem restricted, given the limited sample size. The immunohistochemical staining characteristics of endothelial cells in the pathology of the 9 patients presented in the department align with the pathological features of EHE: epithelioid cells arranged in cords and nests within the stroma but do not exhibit vasoformation. The pathological finding of poorly formed endothelial cells is the primary criterion for identifying EHE, but It is also proved that immunohistochemistry for CAMTA1 expression of nuclear is a significant method to distinguish EHE from other epithelioid vascular tumors including epithelioid angiosarcoma, epithelioid sarcoma which have the mimic histologic features with the expression of TFE3 by immunohistochemistry being another candidate method (30, 31).

In one study aiming at detecting common secondary genomic variants associated with advanced EHE from 49 participants, more than half patients exhibited pathogenic genomic variants in addition to TAZ-CAMTA1 fusion and 18.4% patients in the study showed potentially targetable genomic variants. Importantly, patients who were older were more likely to have clinically targetable variants and the same condition occurred in patients with III/IV stage (32). The aforementioned literature emphasizes that secondary mutations may be the reason for the transition of EHE from indolent to malignant. It also points out that as age increases and disease stage advances, there may be a higher likelihood of secondary mutations occurring, leading to potentially more uncontrollable disease progression. This is consistent with the impact observed in the analysis of the machine results we examined.

It is unknown why the chemotherapy exhibits opposite effect in our Cox Model. Several reasons might hide behind the above question. First of all, the SEER database lacks comprehensive specific information regarding chemotherapy. It is not clear which chemotherapy agent is used for EHE patients. Secondly, the progress of chemotherapy seemed stagnant before the mechanism of EHE was discovered and the oral drug therapy is limited for the rarity of EHE. Conventional chemotherapy such as anthracyclines regimens、pazopanib、paclitaxel and so on exerted restricted effect on treatment of HEHE (33). Thirdly, the information collected in the SEER database spans a wide range of years, and until recent clinical trials have shown significant efficacy of IFN-a 2b (34), Anti-VEGF chemotherapeutic agents such as bevacizumab, pazopanib, sorafenib thalidomide (35–39) and mTOR inhibitor sirolimus(rapamycin) (20, 40–42) for patients, the effectiveness of chemotherapy in treating EHE patients remained uncertain. By the way, it is hard to explain why the sequence numbers which are all reportable neoplasms over the lifetime of the patient have the positive correlation with survival times.

The use of surgical treatment and surgical types should be considered exhaustively according to the tumors location within the liver、the size of the mass、number of nodules、the status of vascular invasion、condition of extrahepatic diseases (22). Patients who underwent surgical treatments had significantly higher survival than those did nothing, and multivariate analysis revealed surgical therapy was only independent prognostic factor for survival (7). Group analysis can provide a new understanding of the suitability for surgical treatment, specifically identifying when surgery is most beneficial. For example, as mentioned earlier, in cases where the tumor is in a more advanced stage the potential benefits of surgery may be limited. Surgical treatments mainly consist of surgical resection and liver transplantation (LT). There had concluded that over half patients benefited from surgical resection and LT also shows excellent 5-year survival outcome in diverse clinical trials (9, 22, 43–47). some articles summarized that surgical resection had better overall survival rates and higher disease-free-survival than LT (9, 20, 48) while others demonstrated that there was no significantly difference between two modalities (5). To sum up, the choice of surgical resection and liver transplantation should be considered carefully after exact analysis of both benefits and risks and group analysis may offer some valuable insights for surgical decision-making.

There are several limitations in the study. Firstly, in the SEER data, radiation therapy and chemotherapy are all treated as binary variables, and the ‘stage’ variable is categorized as distant, localized, and regional, which may be somewhat generalized and lack specificity. Additionally, as mentioned earlier, the chemotherapy information is limited and outdated, and the study findings may be influenced by a lag in recording research advancements. Furthermore, some of the subgroup analyses lack convincing power due to small sample sizes. Lastly, the dataset from our department is limited in size. While the clinical symptoms, imaging information, and pathological features are somewhat representative, it lacks the generality of larger sample sizes.

Recent advancements in fundamental research and omics analyses are shedding light on EHE’s complexities. One study identified potentially targetable genomic variants in EHE, emphasizing variants like CDKN2A/B, which are notably involved in cell cycle regulation and DNA damage repair (32). Notably, the loss of CDKN2A/B was prevalent in older patients and was linked to more aggressive EHE behavior (49). Investigations also revealed that fusion proteins in EHE can modulate the chromatin environment and hyperactivate a TEAD-based transcriptional program (50). Single-cell RNA sequencing (scRNA-seq) highlighted EHE’s cellular heterogeneity, suggesting potential underlying pathways that merit further exploration (51, 52). While anti-VEGF therapies and mTOR inhibitors have clinical implications, MEK inhibitors and YAP/TAZ-TEAD disruptors have shown promise in reducing EHE cell proliferation, although their clinical efficacy remains to be ascertained (53–56).

## 5 Conclusions

Machine learning and Cox regression models have highlighted the significant importance of surgical treatment for HEHE. Given the limited basic research and the lack of further clinical translation for HEHE, surgical treatment remains a worthwhile and preferred option for consideration.

## Data Availability

The original contributions presented in the study are included in the article/**Supplementary Materials**, further inquiries can be directed to the corresponding authors.
