# Supplementary material for: Comprehensive evaluation of clinical outcomes in hepatic epithelioid hemangioendothelioma subsets: insights from SEER Database and departmental cohort analysis
Source: Front Immunol. 2024 Oct 22;15:1491922. doi: 10.3389/fimmu.2024.1491922 (PMC11534872; doi:10.3389/fimmu.2024.1491922)
Supplement: Supplementary file 1 [file DataSheet1.docx]

Supplementary Material

**Comprehensive Evaluation of Clinical Outcomes in Hepatic Epithelioid Hemangioendothelioma Subsets: Insights from SEER Database and Departmental Cohort Analysis**

Bingchen Wang1†,Xiao Chen1†,Rongxuan Li1†, Bolun Ai2†, Feng Ye3, Jianjun Zhao 1, Yefan Zhang1, Zhen Huang1, Zhiyu Li1, Xinyu Bi1,Hong Zhao1, Dayong Cao1*,Jianqiang Cai1* , Jianguo Zhou1*, Tao Yan4*

**^*^Correspondence:
Dayong Cao1***

**caodayongdoctty@163.com,**

**Jianqiang Cai1***

**caijianqiangdoctty@163.com**

**Jianguo Zhou1***

**zjgtydoct@163.com**

**Tao Yan4***

**blizzardyt@163.com**

**Supplementary Table 1.**

| Supplementary Table 1. | **test** | **train** | **p.overall** |
| --- | --- | --- | --- |
|  | ***N=66*** | ***N=247*** |  |
| Age | 55.0 [37.5;64.0] | 52.0 [39.0;65.0] | 0.716 |
| Sex: |  |  | 0.593 |
| Male | 33 (50.0%) | 112 (45.3%) |  |
| Female | 33 (50.0%) | 135 (54.7%) |  |
| Year_of_diagnosis: |  |  | 1.000 |
| before and in 2010 | 30 (45.5%) | 113 (45.7%) |  |
| after 2010 | 36 (54.5%) | 134 (54.3%) |  |
| Race: |  |  | 0.088 |
| White | 48 (72.7%) | 205 (83.0%) |  |
| others | 18 (27.3%) | 42 (17.0%) |  |
| Combined_Summary_Stage: |  |  | 0.810 |
| Localized | 19 (28.8%) | 70 (28.3%) |  |
| Regional | 11 (16.7%) | 35 (14.2%) |  |
| Distant | 24 (36.4%) | 84 (34.0%) |  |
| Unknown/unstaged | 12 (18.2%) | 58 (23.5%) |  |
| Surg_Prim_Site: |  |  | 0.201 |
| no surgery or unknown | 50 (75.8%) | 179 (72.5%) |  |
| Wedge or segmental resection or Lobectomy | 14 (21.2%) | 44 (17.8%) |  |
| Hepatectomy and or transplant | 2 (3.03%) | 24 (9.72%) |  |
| Radiation_recode: |  |  | 0.472 |
| None or Unknown | 65 (98.5%) | 236 (95.5%) |  |
| radiation performed | 1 (1.52%) | 11 (4.45%) |  |
| Chemotherapy_recode: |  |  | 0.057 |
| No or Unknown | 41 (62.1%) | 185 (74.9%) |  |
| Yes | 25 (37.9%) | 62 (25.1%) |  |
| Systemic_Sur_Seq: |  |  | 0.565 |
| no Systemic therapy after and before surgery | 61 (92.4%) | 233 (94.3%) |  |
| Systemic therapy after or before surgery | 5 (7.58%) | 14 (5.67%) |  |
| Sequence_number: |  |  | 0.278 |
| One primary only | 50 (75.8%) | 204 (82.6%) |  |
| Over one | 16 (24.2%) | 43 (17.4%) |  |
| Median_household_income_inflation_adj_to_2021: |  |  | 1.000 |
| below $70000 | 32 (48.5%) | 119 (48.2%) |  |
| more than $70000 | 34 (51.5%) | 128 (51.8%) |  |
| Rural_Urban_Continuum_Code: |  |  | 0.172 |
| Metropolitan (1 million+) | 51 (77.3%) | 167 (67.6%) |  |
| Other metropolitan or non-metropolitan | 15 (22.7%) | 80 (32.4%) |  |

**Supplementary Table 2.**

| Supplementary Table 2 | **no surgery or unknown** | **Surgery performed** | **p.overall** |
| --- | --- | --- | --- |
|  | ***N=66*** | ***N=66*** |  |
| Age | 51.6 (19.2) | 51.4 (15.6) | 0.953 |
| Sex: |  |  | 1.000 |
| Male | 29 (43.9%) | 30 (45.5%) |  |
| Female | 37 (56.1%) | 36 (54.5%) |  |
| Year_of_diagnosis: |  |  | 0.860 |
| before and in 2010 | 27 (40.9%) | 29 (43.9%) |  |
| after 2010 | 39 (59.1%) | 37 (56.1%) |  |
| Race: |  |  | 1.000 |
| White | 56 (84.8%) | 56 (84.8%) |  |
| others | 10 (15.2%) | 10 (15.2%) |  |
| Combined_Summary_Stage: |  |  | 0.258 |
| Localized | 31 (47.0%) | 36 (54.5%) |  |
| Regional | 18 (27.3%) | 10 (15.2%) |  |
| Distant | 9 (13.6%) | 14 (21.2%) |  |
| Unknown/unstaged | 8 (12.1%) | 6 (9.09%) |  |
| Radiation_recode: |  |  | 1.000 |
| None or Unknown | 62 (93.9%) | 62 (93.9%) |  |
| radiation performed | 4 (6.06%) | 4 (6.06%) |  |
| Chemotherapy_recode: |  |  | 0.259 |
| No or Unknown | 51 (77.3%) | 57 (86.4%) |  |
| Yes | 15 (22.7%) | 9 (13.6%) |  |
| Systemic_Sur_Seq: |  |  | 1.000 |
| no Systemic therapy after and before surgery | 62 (93.9%) | 62 (93.9%) |  |
| Systemic therapy after or before surgery | 4 (6.06%) | 4 (6.06%) |  |
| Sequence_number: |  |  | 0.839 |
| One primary only | 51 (77.3%) | 49 (74.2%) |  |
| Over one | 15 (22.7%) | 17 (25.8%) |  |
| Median_household_income_inflation_adj_to_2021: |  |  | 0.601 |
| below $70000 | 34 (51.5%) | 30 (45.5%) |  |
| more than $70000 | 32 (48.5%) | 36 (54.5%) |  |
| Rural_Urban_Continuum_Code: |  |  | 0.848 |
| Metropolitan (1 million+) | 46 (69.7%) | 48 (72.7%) |  |
| Other metropolitan or non-metropolitan | 20 (30.3%) | 18 (27.3%) |  |
| status | 0.55 (0.50) | 0.24 (0.43) | <0.001 |
| time | 4.67 (4.83) | 6.66 (5.17) | 0.024 |

**Supplementary Table 3.**

| Supplementary Table 3 Dependent: Surv(time, status == 1) | | all | HR (univariable) | HR (multivariable) | HR (final) |
| --- | --- | --- | --- | --- | --- |
| Age | Mean ± SD | 51.5 ± 17.5 | 1.02 (1.00-1.03, p=.089) |  |  |
| Sex | Male | 59 (44.7%) |  |  |  |
|  | Female | 73 (55.3%) | 0.75 (0.44-1.30, p=.308) |  |  |
| Year_of_diagnosis | before and in 2010 | 56 (42.4%) |  |  |  |
|  | after 2010 | 76 (57.6%) | 0.68 (0.38-1.20, p=.180) |  |  |
| Race | White | 112 (84.8%) |  |  |  |
|  | others | 20 (15.2%) | 0.90 (0.40-1.99, p=.792) |  |  |
| Combined_Summary_Stage | Localized | 67 (50.8%) |  |  |  |
|  | Regional | 28 (21.2%) | 1.58 (0.75-3.32, p=.227) | 1.11 (0.52-2.37, p=.781) | 1.11 (0.52-2.37, p=.781) |
|  | Distant | 23 (17.4%) | 2.91 (1.43-5.91, p=.003) | 2.18 (0.95-5.01, p=.066) | 2.18 (0.95-5.01, p=.066) |
|  | Unknown/unstaged | 14 (10.6%) | 2.65 (1.18-5.96, p=.018) | 2.64 (1.16-5.98, p=.020) | 2.64 (1.16-5.98, p=.020) |
| Surg_Prim_Site | no surgery or unknown | 66 (50.0%) |  |  |  |
|  | Surgery performed | 66 (50.0%) | 0.35 (0.19-0.63, p<.001) | 0.31 (0.17-0.57, p<.001) | 0.31 (0.17-0.57, p<.001) |
| Chemotherapy_recode | No or Unknown | 108 (81.8%) |  |  |  |
|  | Yes | 24 (18.2%) | 2.40 (1.32-4.38, p=.004) | 2.04 (0.96-4.36, p=.064) | 2.04 (0.96-4.36, p=.064) |
| Sequence_number | One primary only | 100 (75.8%) |  |  |  |
|  | Over one | 32 (24.2%) | 0.99 (0.53-1.86, p=.982) |  |  |
| Median_household_income_inflation_adj_to_2021 | below $70000 | 64 (48.5%) |  |  |  |
|  | more than $70000 | 68 (51.5%) | 0.59 (0.34-1.03, p=.062) |  |  |
| Rural_Urban_Continuum_Code | Metropolitan (1 million+) | 94 (71.2%) |  |  |  |
|  | Other metropolitan or non-metropolitan | 38 (28.8%) | 1.89 (1.08-3.29, p=.025) | 2.13 (1.20-3.79, p=.010) | 2.13 (1.20-3.79, p=.010) |
| n=132, events=52, Likelihood ratio test=33.63 on 6 df(p<.001) | | | | | |

**Supplementary Table 4.**

| Supplementary Table 4 | **Resection or Lobectomy** | **Hepatectomy or transplant** | **p.overall** |
| --- | --- | --- | --- |
|  | ***N=58*** | ***N=26*** |  |
| Age | 50.2 (17.0) | 46.5 (12.0) | 0.257 |
| Sex: |  |  | 0.194 |
| Male | 23 (39.7%) | 15 (57.7%) |  |
| Female | 35 (60.3%) | 11 (42.3%) |  |
| Year_of_diagnosis: |  |  | 0.330 |
| before and in 2010 | 23 (39.7%) | 14 (53.8%) |  |
| after 2010 | 35 (60.3%) | 12 (46.2%) |  |
| Race: |  |  | 1.000 |
| White | 48 (82.8%) | 22 (84.6%) |  |
| others | 10 (17.2%) | 4 (15.4%) |  |
| Combined_Summary_Stage: |  |  | 0.007 |
| Localized | 34 (58.6%) | 6 (23.1%) |  |
| Regional | 9 (15.5%) | 12 (46.2%) |  |
| Distant | 11 (19.0%) | 6 (23.1%) |  |
| Unknown/unstaged | 4 (6.90%) | 2 (7.69%) |  |
| Radiation_recode: |  |  | 0.584 |
| None or Unknown | 56 (96.6%) | 24 (92.3%) |  |
| radiation performed | 2 (3.45%) | 2 (7.69%) |  |
| Chemotherapy_recode: |  |  | 0.140 |
| No or Unknown | 48 (82.8%) | 17 (65.4%) |  |
| Yes | 10 (17.2%) | 9 (34.6%) |  |
| Systemic_Sur_Seq: |  |  | 0.216 |
| no Systemic therapy after and before surgery | 50 (86.2%) | 19 (73.1%) |  |
| Systemic therapy after or before surgery | 8 (13.8%) | 7 (26.9%) |  |
| Sequence_number: |  |  | 0.889 |
| One primary only | 47 (81.0%) | 20 (76.9%) |  |
| Over one | 11 (19.0%) | 6 (23.1%) |  |
| Median_household_income_inflation_adj_to_2021: |  |  | 1.000 |
| below $70000 | 26 (44.8%) | 12 (46.2%) |  |
| more than $70000 | 32 (55.2%) | 14 (53.8%) |  |
| Rural_Urban_Continuum_Code: |  |  | 0.348 |
| Metropolitan (1 million+) | 37 (63.8%) | 20 (76.9%) |  |
| Other metropolitan or non-metropolitan | 21 (36.2%) | 6 (23.1%) |  |


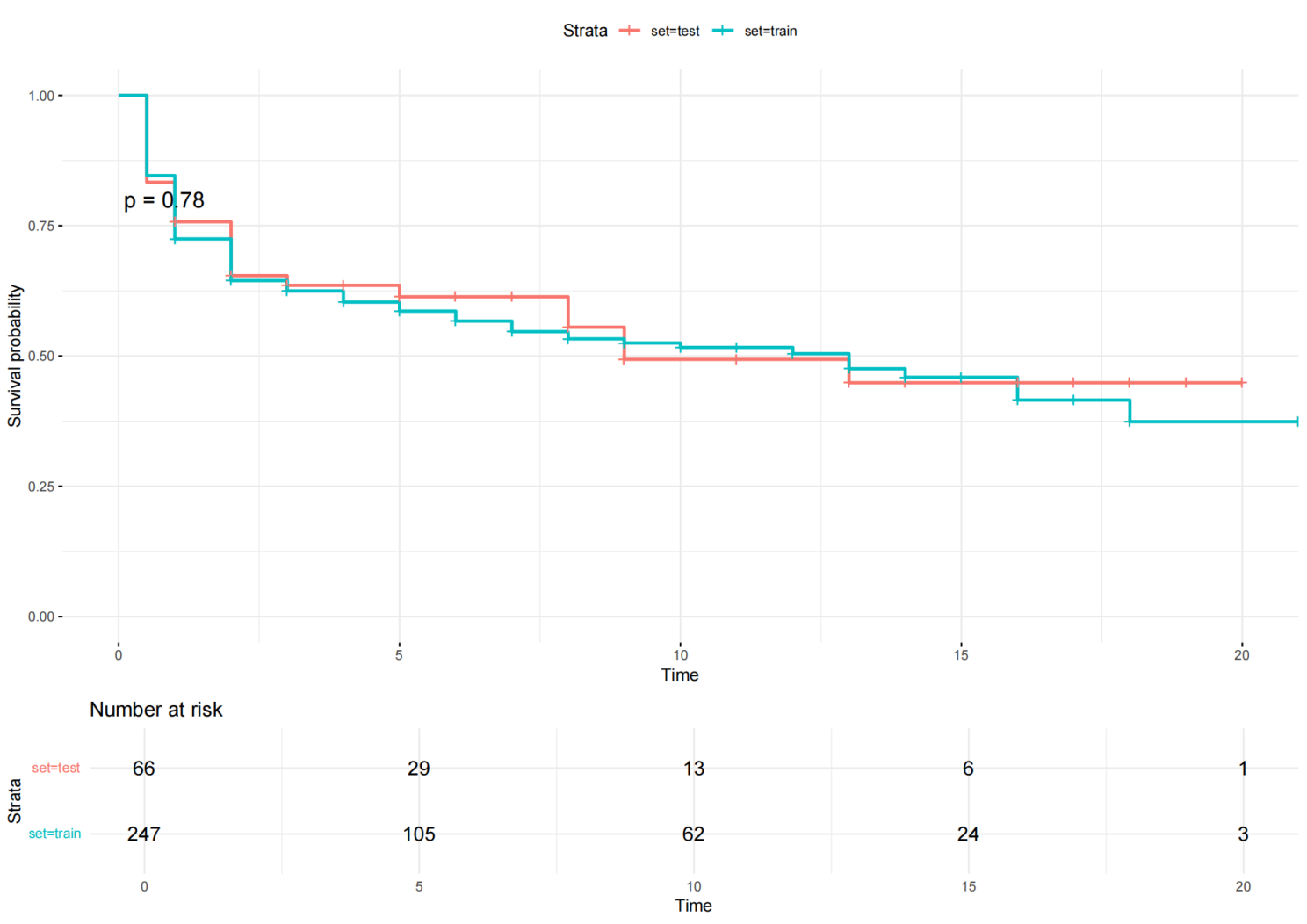


**Supplementary Figure 1.** Comparison of the Kaplan-Meier curves between the train set and test set .


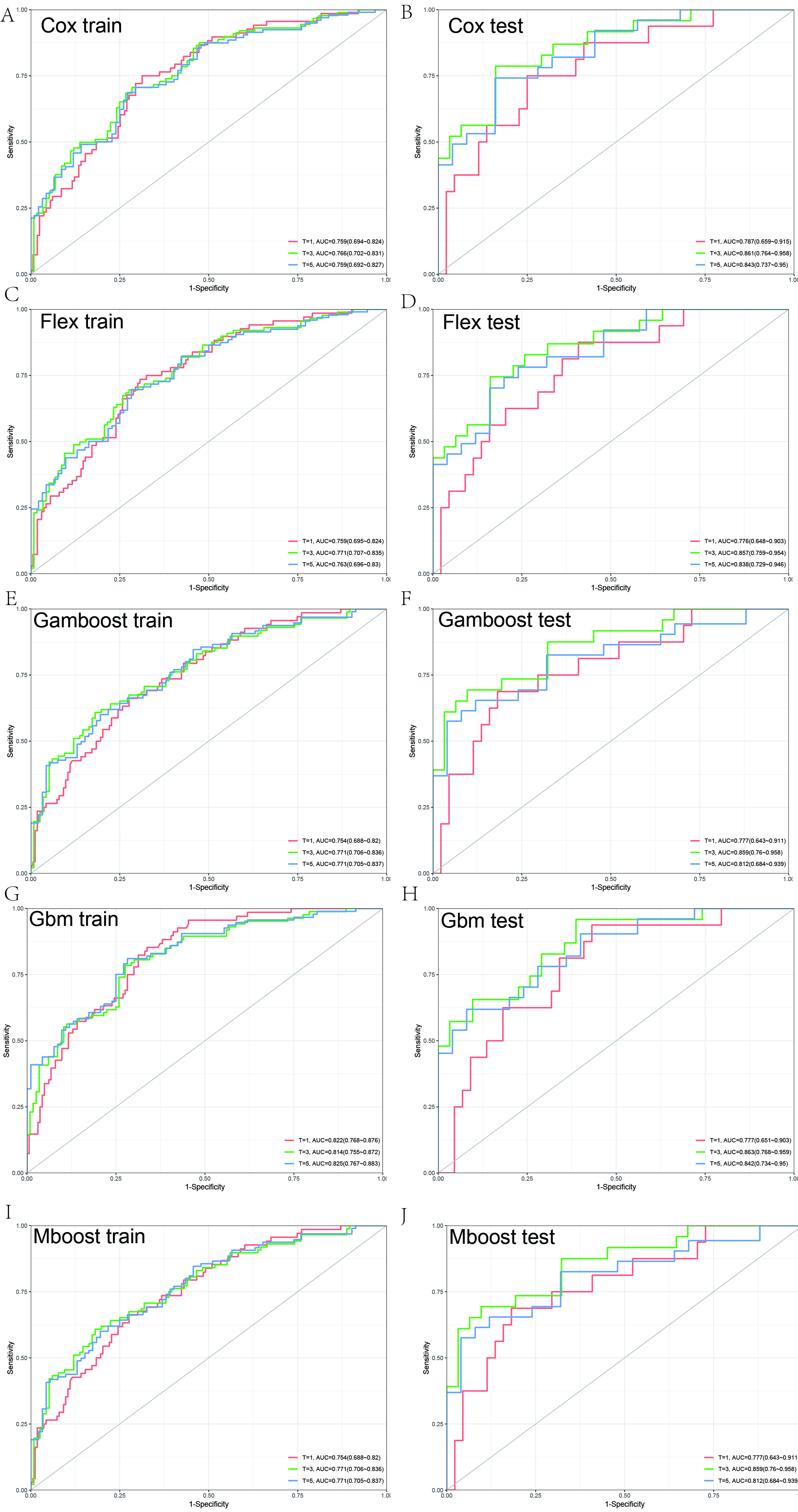


**Supplementary Figure 2**. Receiver operating characteristic curves of **(A)** cox train model , **(B)** cox test model, **(C)** flexible train model, **(D)** flexible test model, **(E)** Gamboost train model, **(F)** Gamboost test model,**(G)** Gbm train model, **(H)** Gbm test model, **(I)** Mboost train model, **(J)** Mboost test model
